# A multicriteria decision making approach applied to improving maintenance policies in healthcare organizations

**DOI:** 10.1186/s12911-016-0282-7

**Published:** 2016-04-23

**Authors:** María Carmen Carnero, Andrés Gómez

**Affiliations:** Business Administration Department, University of Castilla-La Mancha, Avda. Camilo José Cela s/n, 13071 Ciudad Real, Spain; Centre for Management Studies (CEG-IST), University of Lisbon, Instituto Superior Tecnico, Lisbon, Portugal; SESCAM, University General Hospital of Ciudad Real, C/Obispo Rafael Torija s/n, 13005 Ciudad Real, Spain

**Keywords:** Maintenance policies, Multicriteria analysis, MACBETH, Decision-making techniques, Markov chains, Healthcare organizations, Dialysis subsystems

## Abstract

**Background:**

Healthcare organizations have far greater maintenance needs for their medical equipment than other organization, as many are used directly with patients. However, the literature on asset management in healthcare organizations is very limited. The aim of this research is to provide more rational application of maintenance policies, leading to an increase in quality of care.

**Methods:**

This article describes a multicriteria decision-making approach which integrates Markov chains with the multicriteria Measuring Attractiveness by a Categorical Based Evaluation Technique (MACBETH), to facilitate the best choice of combination of maintenance policies by using the judgements of a multi-disciplinary decision group. The proposed approach takes into account the level of acceptance that a given alternative would have among professionals. It also takes into account criteria related to cost, quality of care and impact of care cover.

**Results:**

This multicriteria approach is applied to four dialysis subsystems: patients infected with hepatitis C, infected with hepatitis B, acute and chronic; in all cases, the maintenance strategy obtained consists of applying corrective and preventive maintenance plus two reserve machines.

**Conclusions:**

The added value in decision-making practices from this research comes from: (i) integrating the use of Markov chains to obtain the alternatives to be assessed by a multicriteria methodology; (ii) proposing the use of MACBETH to make rational decisions on asset management in healthcare organizations; (iii) applying the multicriteria approach to select a set or combination of maintenance policies in four dialysis subsystems of a health care organization. In the multicriteria decision making approach proposed, economic criteria have been used, related to the quality of care which is desired for patients (availability), and the acceptance that each alternative would have considering the maintenance and healthcare resources which exist in the organization, with the inclusion of a decision-making group. This approach is better suited to actual health care organization practice and depending on the subsystem analysed, improvements are introduced that are not included in normal maintenance policies; in this way, not only have different maintenance policies been suggested, but also alternatives that, in each case and according to viability, provide a more complete decision tool for the maintenance manager.

## Background

Healthcare organizations have special characteristics which distinguish them from other companies, such as the existence and constant renovation of a wide variety of high-tech equipment for diagnosis and treatment (electromedical and biomedical systems) [1–3], alongside conventional low-tech facilities [4], human resources with varied training and much higher maintenance requirements for apparatus and facilities, as many medical devices are used directly on patients [5].

The influence of this high-tech medical equipment on treatment, diagnostics and patient safety is well understood [6]; however, the importance of maintenance for this medical technology is not so well known [7]. This is because maintenance is considered a support service. In fact, the literature on asset management in healthcare organizations is very limited.

Nonetheless, the quality of maintenance affects the availability and working of medical equipment, and also the safety of patients and of the healthcare workers using that equipment. That is, the quality of maintenance is crucial to the standard of healthcare provided to patients [8]; another point is that significant economic resources are wasted by poor or ineffective maintenance [9, 10]. It is also true that excellent asset and facility management practices are essential in guaranteeing sustainable development based on eco-efficiency and reduction of environmental impact [11]. This factor is key in healthcare organizations, which are typically large consumers of energy and water.

Almeida and Bohoris (1995) [12] and Martorell et al. (2005) [13] underline the goodness of multicriteria techniques in the area of maintenance, especially with problems involving reliability, maintainability, availability and safety. The choice of maintenance policy is a complex decision since it needs to bring together strategic questions which link up with maintenance strategy and so with business strategy, and technical questions surrounding, for example, the kinds of failure in the machines. It is, therefore, a decision that requires profound thought, as a number of different qualitative and quantitative criteria must be considered [14], which justifies the use of multicriteria techniques.

The literature shows how multicriteria techniques have been successfully applied to choosing maintenance policies or strategies for the oil and gas industries [15–17], paper mills and pumping stations [18], the weapons system of the Norwegian Army [19], in manufacturing companies [20–22], an urban waste water treatment plant [23], a petrochemical plant and the food industry [24], a thermal power plant [25], the textile industry [26, 27], aircraft systems [28], a chemical plant [29], a processing plant [30], the railway industry [31], a newspaper printing facility [32], a mining company [33], a dump truck [34], a steel company [35], and a cogeneration system for an ethanol and sugar plant [36].

None of these studies involved a health care organization. Only Taghipour et al. (2011) [37] use the Analytic Hierarchy Process (AHP) to prioritize medical devices according to their criticality. These criticality score values can be used to establish guidelines for selecting appropriate maintenance strategies for different classes of devices. Function, mission criticality, age, risk, recalls and hazard alerts and maintenance requirements are the criteria used to determine the criticality score value. The existence of this single precedent shows the lack of importance traditionally given to support processes in hospitals.

This research may therefore may assist those in charge of the maintenance departments or technical services of health care organizations in their decision making processes; but it could also guide managers of quality departments and hospital managers as to actions for strategic improvement, since it describes a continuous improvement mechanism which can influence the entire organization, and the patients.

Although in reality industrial plants apply combinations of different maintenance policies to assets, in the literature, except in Bertolini and Bevilacqua (2006) [16] and Ghosh and Roy (2010) [30], a single maintenance policy is chosen for each asset; this is worth making clear because the choice of predictive maintenance for a model would eliminate the possibility of applying corrective maintenance, when in real life this does not happen and several maintenance policies are applied together to the same asset.

This study describes a multicriteria decision-making approach to facilitate the choice of a combination of the most suitable maintenance policies for critical medical equipment used directly on patients; furthermore, among the alternative possibilities it considers actions for improvement to increase the reliability of the equipment, such as redundancies in specific medical devices and an increase in spare parts. The multicriteria decision making approach combines Markov chains with the multicriteria Measuring Attractiveness by a Categorical Based Evaluation Technique (MACBETH). The multicriteria model described takes into account the level of acceptance that a given alternative would have among professionals in the field, since they are the ones who will use and maintain the systems, and are best placed to assess the needs of each device. It also takes into account criteria related to cost, quality of care and impact of care cover. The multicriteria approach has been applied to four subsystems of dialysis: patients infected with hepatitis C, infected with hepatitis B, acute and chronic. It also sets out the real implications that applying the results would have for availability and quality of care in a healthcare organization.

Overall, the study shows how to integrate different mathematical techniques in a combination of different maintenance policies, together with decisions about spare parts and facility design, which involves different actors and in which quality of care, understood in this case to be business profitability, is the final aim; all this within the framework of a continuous improvement approach, as well as business process re-engineering as noted by Cigolini et al. (2008) [11].

The main contributions of this research may therefore be summed up as:

1. Integrating the use of Markov chains to obtain the alternatives to be assessed by a multicriteria methodology.
2. Proposing the use of MACBETH to make rational decisions on asset management in healthcare organizations.
3. Applying the multicriteria decision making approach to select a set of combinations of maintenance policies in four dialysis subsystems of a health care organization.

The failure or unavailability of medical equipment can lead to cancellation of diagnostic tests, an increase in the potential risk to the patient, and may even affect people's lives. The research intends, therefore, to make more widely known a decision-making approach which optimizes the application of maintenance policies to improve quality of care.

This article is structured as follows. Firstly, it describes the methodological aspects used in the research relating to the MACBETH method and Markov Chains. Then, it sets out the results obtained by applying Markov chains to the subsystems of dialysis. Next, it presents a multicriteria model for optimizing maintenance policies applied to dialysis subsystems of patients infected with hepatitis C and B, in both acute and chronic patients; it includes the process of structuring and weighting and the possible alternatives. It then shows the results obtained for each subsystem of dialysis, the sensitivity analysis, and it analyses the real implications these results would have on the availability and quality of care. Finally, the conclusions are presented.

## Methods

We shall now describe the methodological aspects of the two techniques to be applied in this research: the multicriteria MACBETH technique, and Markov chains.

### MACBETH Method

Most of the literature on choice of maintenance policy applies the AHP in a crisp or fuzzy framework, although in some cases the Analytic Network Process (ANP), Technique for Order of Preference by Similarity to Ideal Solution (TOPSIS) (crisp o fuzzy), VIKOR, ELimination and Choice Expressing Reality (ELECTRE), are used.

The MACBETH method is used in this research. The mathematical foundations of MACBETH are described in [38] and are updated in [39]. MACBETH is an interactive approach for cardinal measurement that has been validated in numerous real-world applications [40–52]. MACBETH allows a decision maker or decision-adviser group to assess alternatives by making qualitative comparisons relative to the differences in attractiveness across multiple criteria. MACBETH therefore needs only qualitative judgements about the difference of attractiveness between two elements at a time, in order to generate numerical scores for the options in each criterion and to weight the criteria. MACBETH seems particularly suited to the aggregation of assessment criteria when absolute and relative information is required; this allows the alternatives to be assessed considering specific targets [45].

Additionally, for the application of MACBETH there is a user-friendly software M-MACBETH which helps in the implementation of the whole multicriteria evaluation-aiding process. M-MACBETH allows the simulation of challenges due to hesitation in choosing between two or more categories of difference in attractiveness. This is particularly important in group decisions, such as that described in the research, where the assessments were gathered by consensus among three decision makers. Furthermore, M-MACBETH includes tools to check the consistency of the judgements expressed by the decision group automatically and suggests how to resolve inconsistencies if they arise. It also allows for the development of an extensive analysis of the robustness and sensitivity of the model.

The construction of value functions from the two reference levels that have to be defined for each evaluation criterion, leads to a much more objective and accurate assessment of the alternatives. Moreover, the capacity of M-MACBETH to support interactive group learning for a problem, is the main reason leading to the choice of this model. It also allows rankings to be used in the semantic categories when qualitative judgments regarding the difference of attractiveness between options were elicited from the decision maker. This is particularly interesting because it has permitted the inclusion of group uncertainties in the decision process, without having to use a fuzzy multicriteria model, with greater complexity. MACBETH has other advantages in common with other multicriteria methods such as the possibility of including a large number of decision criteria, which may be qualitative or quantitative, easy for the decision group to understand, etc.

For each criterion a descriptor must be constructed or identified. A descriptor is a set of impact levels, which serves to describe plausible impacts of alternatives with respect to a criterion. It is necessary to rank the impact levels in order of decreasing attractiveness [38].

A value function is required to assign value scores to the performance levels of a descriptor relative to the fixed scores of 0 and 100 assigned to the good and neutral reference levels. MACBETH only needs qualitative judgements to provide value functions, overcoming the scepticism caused by the use of numerical judgements [49].

To provide the qualitative judgements the following categories of difference of attractiveness are used: no difference, difference very weak, weak, moderate, strong, very strong and extreme, or a union of two successive categories. Using these categories, a pairwise comparison judgements matrix is created for each criterion. As each judgement is given, M-MACBETH automatically verifies the consistency of the matrix and suggests modifications to the judgements, which help to eliminate the inconsistencies identified.

These judgement matrices are processed in M-MACBETH by means of linear programming, to build a value function for each criterion which assigns value scores to the performance levels of a descriptor, with 0 as the neutral reference level and 100 as the good reference level. The following linear programming problem has to be solved to obtain the value functions [50]:

1 $$ Min\ \left[v\left({x}^{+}\right)-v\left({x}^{-}\right)\right] $$

Subject to

$$ v\left({x}^{\mathit{\hbox{-}}}\right)=\boldsymbol{0}\ \left( arbitrary\  assignment\right) $$

$$ v(x)-v(y)=0,\ \forall\ x,y\ \in {C}_0 $$

$$ v(x)-v(y)\ge i,\ \forall\ x,y\ \in {C}_i{\displaystyle \cup}\dots {\displaystyle \cup}\;{C}_s\  with\ i,s\in \left\{1,\ 2,\ 3,\ 4,\ 5,\left.6\right\}\  and\ i\le s\right. $$

$$ \begin{array}{l}v(x)-v(y)\ge v(w)-v(z)+i-{s}^{\hbox{'}},\ \forall\ x,y\\ {}\kern1em \in {C}_i{\displaystyle \cup}\dots {\displaystyle \cup }{C}_s\  and\ \forall\ w,z\ \in {C}_{i^{\hbox{'}}}{\displaystyle \cup}\dots {\displaystyle \cup }{C}_{s^{\hbox{'}}}\  with\kern0.37em i,s,{i}^{\hbox{'}},{s}^{\hbox{'}}\in \left\{1,\ 2,\ 3,\ 4,\ 5,\left.6\right\}\  and\ i\le s,\ \right.\ {i}^{\hbox{'}}\\ {}\le {s}^{\hbox{'}}\  and\ i>{s}^{\hbox{'}}\end{array} $$

Where *v*(*x*) is the score assigned to element x of X, *x*^+^ is at least as attractive as any other element of X and *x*^−^ is at most as attractive as any other element of X.

This value function has to be discussed to ensure that it adequately represents the relative magnitude of the decision makers’ judgements [51].

The next stage is to weight the criteria. The weightings of the criteria are assessed using the MACBETH weighting procedure. The possibility of an alternative at the neutral level in all the criteria is considered first. The group was asked to qualitatively judge the increase in overall attractiveness provided by a switch from the neutral level to the most attractive impact level, in each of the criteria, using the MACBETH semantic categories. These judgments form the last column of the matrix in Fig. 6. Next, a comparison is made of the extent to which the change from the neutral level to the good level in the first criterion is preferred to the same change in the second. The same comparison is made between the first criterion and the third, and so on. The rest of the matrix is filled by comparing the switches pairwise, using the MACBETH semantic categories.

**Fig. 1:**
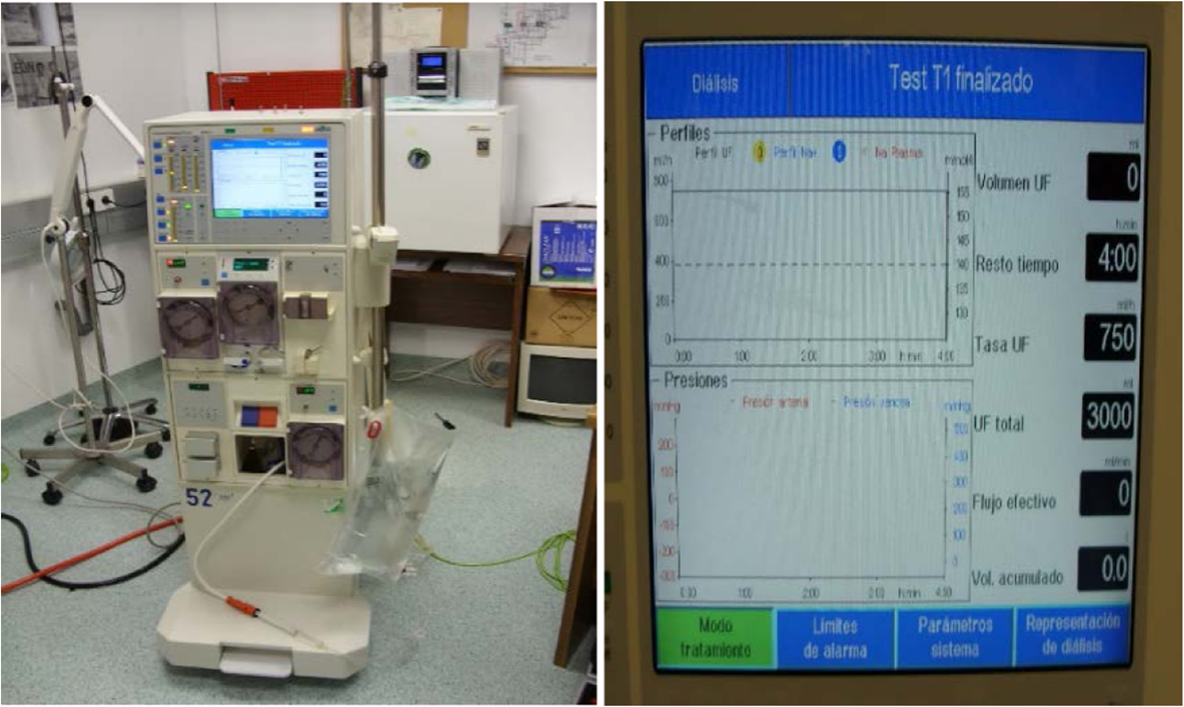
Dialysis subsystems

As in the case of the construction of each value function, the consistency of each judgement included in the matrix is automatically checked. Once the pairwise judgements are made, the decision make is asked to examine and confirm the weights.

The evaluation of an alternative is carried out by simple additive aggregation from bottom to top in the value tree. When considering *n* decision criteria, the performance *V* (*A*) of an alternative *A* is calculated by Equation (2) [52].

2 $$ V(A)={\displaystyle \sum_{i=1}^n{w}_i{v}_i\Big(\mathrm{impact}\ \mathrm{of}}\ A\ \mathrm{on}\ \mathrm{criterion}\kern0.5em i\Big) $$

with ${\displaystyle \sum_{i=1}^n{w}_i=1}$ and *w*_*i*_ > 0 and $$ \left\{\begin{array}{l}{v}_i\;\left(\mathrm{most}\kern0.24em \mathrm{attracive}\kern0.24em \mathrm{impact}\kern0.24em \mathrm{level}\kern0.24em \mathrm{on}\kern0.24em i=100\right.\\ {}{v}_i\;\left(\mathrm{least}\kern0.24em \mathrm{attracive}\kern0.24em \mathrm{impact}\kern0.24em \mathrm{level}\kern0.24em \mathrm{on}\kern0.24em i=0\right.\end{array}\right. $$

and *w*_*i*_ are the relative weights of criteria and *v*_*i*_(impact of *A* on criterion *i*) is the value score of *A* in criterion *i*.

### Markov chains

The Markov chains allow systems to be modelled and their reliability, maintainability, availability and safety parameters to be estimated [53]. For these reasons, it has been widely applied in the literature. For example, calculating the optimal inspection interval by minimizing the expected total cost per time unit [54], to predict the behaviour of repairable elements in the pipes of a nuclear power plant [55], to assess the availability of a system comprising two pumps, one of them actively in reserve [56]; to determine maintenance policies in a catalytic cracking unit [57] or to identify the maximum periodic inspection interval for high tension engines [58].

The modelling of a system by Markov chains consists in obtaining a graph defining the states of the system, and the transition between states is caused by a failure or a repair. Failures can cause the system analysed to break down directly or through wear, in which case the states of decline and wear are considered to be non-catastrophic failure.

A stationary Markov chain in continuous time can be described, as in Hillier and Lieberman (2002) [59]:

- The time it remained in a state *i*, *T*_*i*_, is distributed exponentially with mean *1/q*_*i*_ and failure rate *q*.
- When it leaves a state *i*, the process evolves to a state *j*, with probability *p*_*ij*_, where *p*_*ij*_ satisfies Equation (3).
- 3 $$ \begin{array}{l}{P}_{ij}=0\ \forall i\\ {}{\displaystyle \sum_{j=0}^M{P}_{ij}=1}\ \forall i\end{array} $$
- The next state it reaches after *i* is independent of the time it spent in state *i*.

In continuous time, to describe the transition between states the duration and transition rates [60] must be defined:

4 $$ \begin{array}{l}{q}_{ii}=\underset{\varDelta t\to 0}{Lim}\left\{\left({p}_{ii}\left(\varDelta t\right)-1\right)/\varDelta t\right\}=-{\displaystyle \sum_{i\ne j}{q}_{ij}}\\ {}{q}_{ij}=\underset{\varDelta t\to 0}{Lim}\left\{{p}_{ij}\left(\varDelta t\right)/\varDelta t\right\},\kern1em i\ne j\end{array} $$

Where *q*_*ii*_ the rate of duration in state *i* and *q*_*ij*_ the rate of transition of state *i* to *j. q*_*ii*_ is negative because the probability of remaining in the same state decreases as *t* increases and, *q*_*ij*_ is positive because the probability of changing state increases with *t*.

The matrix *Q* of the transition rate or infinitesimal generator of the chain is defined from Equation (5).

5 $$ Q=\left(\begin{array}{ccc}\hfill {q}_{11}\hfill & \hfill {q}_{12}\hfill & \hfill \dots \hfill \\ {}\hfill {q}_{21}\hfill & \hfill {q}_{22}\hfill & \hfill \dots \hfill \\ {}\hfill \dots \hfill & \hfill \dots \hfill & \hfill \dots \hfill \end{array}\right)=\underset{\varDelta t\to 0}{Lim}\frac{P\left(\varDelta t\right)-I}{\varDelta t} $$

satisfying $$ {\displaystyle \sum_j{q}_{ij}=0} $$.

With respect to the probabilities of the stable state, the continuous-time transition equations satisfy the Chapman-Kolmogorov equations (Equation (6)) [61].

6 $$ {p}_{ij}(t)={\displaystyle \sum_{k=1}^M{p}_{ik}\left(t-s\right)}\kern0.5em {p}_{kj}(s)\kern0.75em \forall i,j\kern0.5em 0\le s\le t $$

where:

$$ {p}_{ij}\left(t+\varDelta t\right)-{p}_{ij}={\displaystyle \sum_k\left\{{p}_{ik}\left(t+\varDelta t-s\right)-{p}_{ik}\left(t-s\right)\right\}}\kern0.75em {p}_{kj}(s) $$

$$ with\ s\to t,\varDelta t\to 0:{p}_{ik}\left(t-s\right)\to 0,i\ne k;{p}_{ii}\left(t-s\right)\to 1,\ \mathrm{is}\ \mathrm{obtained} $$

7 $$ d{p}_{ij}(t)/dt={\displaystyle \sum_k{q}_{ik}}{p}_{kj}(t) $$

and in matrix form:

8 $$ \begin{array}{l}dP(t)/dt=P(t)Q\ \\ {}{\left[dP(t)/dt\right]}^T={Q}^TP{(t)}^T\end{array} $$

On the other hand, the probability of being in state *i* at instant *t* is:

9 $$ {\pi}_i(t)=P\left\{X(t)=i\right\}={\displaystyle \sum_kP\left\{X(t)=i\left|X(0)=k\right.\right\}}P\left\{X(0)=k\right\}={\displaystyle \sum_k{p}_{ki}(t){\pi}_k(0)} $$

and in matrix form:

10 $$ \pi (t)=\pi (0)P(t) $$

There is assumed to be a limit:

11 $$ \underset{t\to \infty }{Lim}\pi (t)=\pi =\pi (0)P\left(\infty \right) $$

For stable Markov chains the following is satisfied

12 $$ \underset{t\to \infty }{Lim}dP(t)/dt=0 $$

and substituting into Equation (8) gives:

13 $$ P\left(\infty \right)Q=0 $$

right-multiplying Equation (11) by *Q* in both terms and substituting into Equation (13) gives,

14 $$ \pi Q=\pi (0)P\left(\infty \right)Q=0 $$

On the other hand, it is clear that,

15 $$ {\displaystyle \sum {\pi}_j}=1 $$

The system of equations (14) is usually presented according to Equation (16),

16 $$ {Q}^T{\pi}^T={0}^T $$

where *π*^*T*^ and *Q*^*T*^ are column vectors.

Substituting Equation (15) into Equation (16) with the aim of achieving a non-homogeneous system of equations gives Equation (17).

17 $$ \left(\begin{array}{cccc}\hfill {q}_{11}\hfill & \hfill {q}_{21}\hfill & \hfill \dots \hfill & \hfill {q}_{n1}\hfill \\ {}\hfill {q}_{12}\hfill & \hfill {q}_{22}\hfill & \hfill \dots \hfill & \hfill {q}_{n2}\hfill \\ {}\hfill \dots \hfill & \hfill \dots \hfill & \hfill \dots \hfill & \hfill \dots \hfill \\ {}\hfill 1\hfill & \hfill 1\hfill & \hfill \dots \hfill & \hfill 1\hfill \end{array}\right)\left(\begin{array}{c}\hfill {\pi}_1\hfill \\ {}\hfill {\pi}_2\hfill \\ {}\hfill \dots \hfill \\ {}\hfill {\pi}_n\hfill \end{array}\right)=\left(\begin{array}{c}\hfill 0\hfill \\ {}\hfill 0\hfill \\ {}\hfill \dots \hfill \\ {}\hfill 1\hfill \end{array}\right) $$

The processes analysed in this study show the following characteristics:

- A state may change at any time.
- The number of states a system may reach is finite.
- The life period studied is, within the life cycle, that of constant failure rate; the same concept is used for repairs. This means that the failure and repair distribution is exponential.

The subsystems to be analysed behave like stable Markov chains in continuous time, because at any instant there can be a change of state (failure or startup); it is considered that in the transition time used to go from one state to another, ∆t, there can only be one failure or repair, not necessarily consecutive.

The Markov model assesses the probability of moving from a known state, according to the configuration of the system studied, to another, passing through the dependencies between them, whether failures or repairs.

To study the development of reliability and availability of systems and therefore to predict their behaviour using Markov chains, two systems with n + 1 possible states are considered, such that each state represents a level of wear, with k the maximum number of poor-quality states that will still allow the system to function.

Each level of wear can be identified by the number of elements that are out of order, although this can mean a progressive deterioration in the system on a set scale, such as for example the progressive wear on systems of pipes, or conduits in general, or a machine with wear due to friction or rust.

Thus, the following states can be defined [62]:

- State 0. The systems is working perfectly.
- State 1. One of the elements is not working or the system is at level one of deterioration.
- State 2. Two of the elements are not working or the system is at level two of deterioration.
- State *k. k* elements are not working or the system is at level k of deterioration.
- State *m-1. m-1* elements are not working or the system is at level m-1 of deterioration.
- State *m*. All the elements have failed or the system is completely deteriorated.

A system can move from one state to another according to the connection between them.

Each element of matrix *Q*^*T*^ is given by Equation (18) [59]:

18 $$ {p}_{ii}\left(\varDelta t\right)=1-\left\{{\displaystyle \sum_{j=i+1}^m{p}_{ij}\left(\varDelta t\right)+{\displaystyle \sum_{j=1}^{i-1}{p}_{ij}\left(\varDelta t\right)}}\right\} $$

where *P*_*ij*_(*Δt*) ∀ *j* = (*i* + 1 … *n*) is the unreliability or probability of changing state in some *Δt. P*_*ij*_(*Δt*) ∀ *j* = (*i*, *i* − 1) is the equivalent of maintainability. For an exponential distribution such as the failure and repair density, we get:

19 $$ {p}_{ii}\left(\varDelta t\right)=1-\left\{{\displaystyle \sum_{j=i+1}^m\left(1-{e}^{-{\lambda}_{ij}\varDelta t}\right)+{\displaystyle \sum_{j=1}^{i-1}\left(1-{e}^{-{\mu}_{ij}\varDelta t}\right)}}\right\} $$

Substituting in Equation (4) leads to Equation (20) [59]:

20 $$ {q}_{ii}=-\left\{{\displaystyle \sum_{j=i+1}^m{\lambda}_{ij}+{\displaystyle \sum_{j=1}^{i-1}{\mu}_{ij}}}\right\} $$

Analogously:

21 $$ \begin{array}{l}{p}_{ij}\left(\varDelta t\right)={\lambda}_{ij}\kern1em \forall i<j\\ {}{p}_{ij}\left(\varDelta t\right)={\mu}_{ij}\kern1em \forall i>j\end{array} $$

Substituting the previous equation into the unreliability equation (22) gives the system of equations (23), where ^'^ means derived.

22 $$ F\left({t}_1\right)={\displaystyle {\int}_0^{t_1}f(t)}dt\kern0.62em \mathrm{with}\kern0.5em F(0)=0\kern0.5em \mathrm{and}\kern0.5em F\left(\infty \right)=1 $$

23 $$ \left(\begin{array}{c}\hfill {P}_0^{\hbox{'}}\hfill \\ {}\hfill {P}_1^{\hbox{'}}\hfill \\ {}\hfill .\hfill \\ {}\hfill .\hfill \\ {}\hfill {P}_{m-1}^{\hbox{'}}\hfill \\ {}\hfill {P}_m^{\hbox{'}}\hfill \end{array}\right)=\left(\begin{array}{cccccc}\hfill -{\displaystyle \sum_{j=1}^m\lambda oj}\hfill & \hfill {\mu}_{1,0}\hfill & \hfill .\hfill & \hfill .\hfill & \hfill {\mu}_{m-1,0}\hfill & \hfill {\mu}_{m,0}\hfill \\ {}\hfill {\lambda}_{0,1}\hfill & \hfill -\Big({\displaystyle \sum_{j=2}^m\lambda 1j+{\mu}_{1,0}\Big)}\hfill & \hfill {\mu}_{2,1}\hfill & \hfill .\hfill & \hfill .\hfill & \hfill {\mu}_{m,1}\hfill \\ {}\hfill .\hfill & \hfill .\hfill & \hfill .\hfill & \hfill .\hfill & \hfill .\hfill & \hfill .\hfill \\ {}\hfill .\hfill & \hfill .\hfill & \hfill .\hfill & \hfill .\hfill & \hfill .\hfill & \hfill .\hfill \\ {}\hfill {\lambda}_{0,m-1}\hfill & \hfill {\lambda}_{1,m-1}\hfill & \hfill .\hfill & \hfill .\hfill & \hfill -\left(\lambda n-1,m+{\displaystyle \sum_{j=0}^{m-2}\mu n-1,j}\right)\hfill & \hfill {\mu}_{m,m-1}\hfill \\ {}\hfill {\lambda}_{0,m}\hfill & \hfill {\lambda}_{1,m}\hfill & \hfill .\hfill & \hfill .\hfill & \hfill {\lambda}_{m-1,m}\hfill & \hfill -{\displaystyle \sum_{j=0}^{m-1}\mu n,j}\hfill \end{array}\right)\left(\begin{array}{c}\hfill {P}_0\hfill \\ {}\hfill {P}_1\hfill \\ {}\hfill .\hfill \\ {}\hfill .\hfill \\ {}\hfill {P}_{m-1}\hfill \\ {}\hfill {P}_m\hfill \end{array}\right) $$

Availability in a system of *n* elements under repair depends on the level of state *k* necessary to keep working, where *D*(*t*) = *P*_0_(*t*) + *P*_1_(*t*) + … + *P*_*k*_(*t*).

Mean availability is obtained by substituting the values of matrix (23) into equation (17), which gives equation (24).

24 $$ \left(\begin{array}{cccccc}\hfill -{\displaystyle \sum_{j=1}^m\lambda oj}\hfill & \hfill \mu 10\hfill & \hfill .\hfill & \hfill \mu k0\hfill & \hfill .\hfill & \hfill {\mu}_{m0}\hfill \\ {}\hfill \lambda 01\hfill & \hfill -\Big({\displaystyle \sum_{j=2}^m{\lambda}_{1j}+\mu 10\Big)}\hfill & \hfill .\hfill & \hfill \mu k1\hfill & \hfill {\mu}_{m-1,1}\hfill & \hfill {\mu}_{m,1}\hfill \\ {}\hfill .\hfill & \hfill .\hfill & \hfill .\hfill & \hfill .\hfill & \hfill .\hfill & \hfill .\hfill \\ {}\hfill \lambda 0k\hfill & \hfill \lambda 1k\hfill & \hfill .\hfill & \hfill -\left({\displaystyle \sum_{j=k+1}^m\lambda k,j}+{\displaystyle \sum_{j=0}^{k-1}\mu k,j}\right)\hfill & \hfill {\mu}_{m-1,k}\hfill & \hfill {\mu}_{m,k}\hfill \\ {}\hfill .\hfill & \hfill .\hfill & \hfill .\hfill & \hfill .\hfill & \hfill .\hfill & \hfill .\hfill \\ {}\hfill 1\hfill & \hfill 1\hfill & \hfill .\hfill & \hfill .\hfill & \hfill 1\hfill & \hfill 1\hfill \end{array}\right)\left(\begin{array}{c}\hfill {C}_1\hfill \\ {}\hfill {C}_2\hfill \\ {}\hfill .\hfill \\ {}\hfill {C}_k\hfill \\ {}\hfill .\hfill \\ {}\hfill {C}_m\hfill \end{array}\right)=\left(\begin{array}{c}\hfill 0\hfill \\ {}\hfill 0\hfill \\ {}\hfill .\hfill \\ {}\hfill .\hfill \\ {}\hfill .\hfill \\ {}\hfill 1\hfill \end{array}\right) $$

For *k* stable states necessary for the system to work, mean availability *D*_*m*_ is:

25 $$ {D}_m={C}_1+{C}_2 + \dots +{C}_k $$

where *C*_*i*_ is the coefficient obtained from the solution of the previously mentioned system of equations for each alternative analysed.

#### A multicriteria decision making approach to improve maintenance policies on the dialysis subsystems

The University General Hospital of Ciudad Real (UGHCR) is a public hospital belonging to the Castilla-La Mancha Health Service, (SESCAM) which became operational in 2005. It offers a wide range of service and 2700 staff across many categories in both healthcare and non-care roles. It serves 174,550 people directly, and 370,000 potentially, who may be referred from other centres in the region. It sets a regional standard for the specialities of nuclear medicine, eating disorders and its blood bank.

The UGHCR has 2700 machines registered, which include high-tech equipment and facilities in all branches of bio-engineering (gamma cameras, linear accelerator, CAT simulator, PET-CAT, NMR, etc.) as well as conventional equipment and facilities.

In a prior study, the care equipment was structured by subsystems.

### Markov analysis in the dialysis subsystems

The subsystems dedicated to patient dialysis consist of a dialysis machine per patient or position, fed by a network of treated water (see Fig. 1). Each of these has a number of operational and reserve monitors, which are perfectly ready to be used immediately and have almost identical technical characteristics. Table 1 shows the specific characteristics of each dialysis subsystem.

**Fig. 2:**
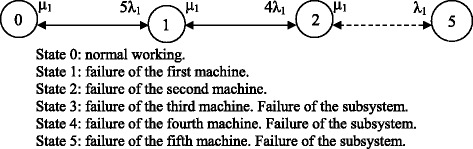
Markov graph for the dialysis subsystem for patients infected with hepatitis C

**Table 1:** Characteristics of the dialysis subsystems

Subsystem	Number of positions for patient care	Number of machines	Failure of the subsystem
Dialysis of patients with hepatitis C	5	7	2 machines out of order
Dialysis of patients with hepatitis B	3	5	2 machines out of order
Dialysis of chronic patients	14	18	4 machines out of order
Dialysis of acute patients	3	5	2 machines out of order

### Dialysis subsystem for patients with hepatitis C

The first subsystem analysed comprises five operational positions for dialysis of infectious hepatitis C patients, and two in reserve; the latter are in perfect working order and may be used at any time, and have similar technical characteristics to the former. The seven dialysis machines share the workload equally, and the time of use is distributed so that they all work the same number of hours per year. Shared use with any other subsystem is not possible. The subsystem is considered to have failed when two machines have broken down.

*λ*_*1*_and *μ*_*1*_ are defined as the failure and repair rates of each monitor when the presence of a maintenance technician is required. *λ*_*2*_ and *μ*_*2*_ are the failure and repair rates of each monitor when the official technical service is contacted. Two alternatives for improving availability are considered, consisting of adding one or two reserve machines to the available set, in the same operating conditions as the others. *D*_*om*_, *D*_*1m*_ and *D*_*2m*_ are defined as the availabilities for seven, eight and nine dialysis machines respectively. Figure 2 shows the resulting Markov graph when the presence of a maintenance technician is required. If the official technical service is contacted, the resulting Markov graph will be the same but characterised by *λ*_*2*_ and *μ*_*2*_.

**Fig. 3:**
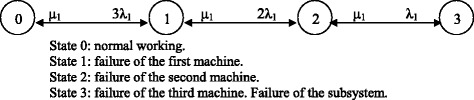
Markov graph for the dialysis subsystem for patients infected with hepatitis B

The 6 × 6 transition matrix of corresponding to the Markov graph in Fig. 2, when the presence of a maintenance technician is required, is shown in Equation (26).

26 $$ {D}_1=\left(\begin{array}{ccccc}\hfill -5{\lambda}_1\hfill & \hfill {\mu}_1\hfill & \hfill .\hfill & \hfill 0\hfill & \hfill 0\hfill \\ {}\hfill 5{\lambda}_1\hfill & \hfill -4{\lambda}_1-{\mu}_1\hfill & \hfill .\hfill & \hfill 0\hfill & \hfill 0\hfill \\ {}\hfill .\hfill & \hfill .\hfill & \hfill .\hfill & \hfill .\hfill & \hfill .\hfill \\ {}\hfill 0\hfill & \hfill 0\hfill & \hfill .\hfill & \hfill -{\lambda}_1-{\mu}_1\hfill & \hfill {\mu}_1\hfill \\ {}\hfill 1\hfill & \hfill 1\hfill & \hfill .\hfill & \hfill 1\hfill & \hfill 1\hfill \end{array}\right) $$

The transition matrix corresponding to the case in which the official technical service is contacted is shown in Equation (27).

27 $$ {D}_1=\left(\begin{array}{ccccc}\hfill -5{\lambda}_2\hfill & \hfill {\mu}_2\hfill & \hfill .\hfill & \hfill 0\hfill & \hfill 0\hfill \\ {}\hfill 5{\lambda}_2\hfill & \hfill -4{\lambda}_2-{\mu}_2\hfill & \hfill .\hfill & \hfill 0\hfill & \hfill 0\hfill \\ {}\hfill .\hfill & \hfill .\hfill & \hfill .\hfill & \hfill .\hfill & \hfill .\hfill \\ {}\hfill 0\hfill & \hfill 0\hfill & \hfill .\hfill & \hfill -{\lambda}_2-{\mu}_2\hfill & \hfill {\mu}_2\hfill \\ {}\hfill 1\hfill & \hfill 1\hfill & \hfill .\hfill & \hfill 1\hfill & \hfill 1\hfill \end{array}\right) $$

The mean availabilities for the original subsystem and for each of the alternatives considered are: *D*_0*m*_ = *C*_0_ + *C*_1_ + *C*_2_, *D*_1*m*_ = *C*_0_ + *C*_1_ + *C*_2_ + *C*_3_ and *D*_2*m*_ = *C*_0_ + *C*_1_ + *C*_2_ + *C*_3_ + *C*_4_; where *C*_*i*_ is the coefficient obtained from the solution of Equation (24) for each alternative analysed.

The failure and repair rates for this subsystem are *λ*_*1*_ = 0.000338 failures/h, *μ*_*1*_ = 0.1 repairs/h, *λ*_*2*_ = 0.000338 failures/h and *μ*_*2*_ = 0.021 repairs/h. That is, as monitors are similar, the failure rates are similar too; the differences are between the repair rates due to the two different situations (the presence of a maintenance technician or contact with the official technical service).

### Dialysis subsystem for patients with hepatitis B

The subsystem for dialysis of patients with hepatitis B consists of three positions with five dialysis machines; the workload is shared among them so that they all work the same number of hours annually. Shared use with any other subsystem is not possible. The subsystem is considered to have failed when two machines have broken down.

*λ*_*1*_ and *μ*_*1*_ are defined as the failure and repair rates of each monitor when the presence of a maintenance technician is required. *λ*_*2*_ and *μ*_*2*_ are the failure and repair rates of each monitor when the official technical service is contacted.

Two alternatives are considered for the improvement of availability, consisting in adding one or two reserve machines to the set available, and sharing the workload proportionally, with the same working conditions as the others. *D*_*om*_, *D*_*1m*_ and *D*_*2m*_ are the availabilities for five, six and seven dialysis machines respectively.

The resulting Markov graph is shown in Fig. 3 when the presence of a maintenance technician is required. If the official technical service is contacted, the resulting Markov graph will be the same but characterised by *λ*_*2*_ and *μ*_*2*_.

**Fig. 4:**
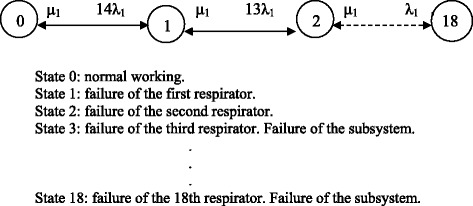
Markov graph for the dialysis subsystem for chronic patients

The 4 × 4 transition matrix corresponding to the Markov graph of Fig. 3, when the presence of a maintenance technician is required, is:

28 $$ {D}_1=\left(\begin{array}{cccc}\hfill -3{\lambda}_1\hfill & \hfill {\mu}_1\hfill & \hfill 0\hfill & \hfill 0\hfill \\ {}\hfill 3{\lambda}_1\hfill & \hfill -2{\lambda}_1-{\mu}_1\hfill & \hfill {\mu}_1\hfill & \hfill 0\hfill \\ {}\hfill 0\hfill & \hfill 2{\lambda}_1\hfill & \hfill -{\lambda}_1-{\mu}_1\hfill & \hfill {\mu}_1\hfill \\ {}\hfill 1\hfill & \hfill 1\hfill & \hfill 1\hfill & \hfill 1\hfill \end{array}\right) $$

The transition matrix corresponding to the case in which the official technical service is contacted is shown in Equation (29).

29 $$ {D}_1=\left(\begin{array}{cccc}\hfill -3{\lambda}_2\hfill & \hfill {\mu}_2\hfill & \hfill 0\hfill & \hfill 0\hfill \\ {}\hfill 3{\lambda}_2\hfill & \hfill -2{\lambda}_2-{\mu}_2\hfill & \hfill {\mu}_2\hfill & \hfill 0\hfill \\ {}\hfill 0\hfill & \hfill 2{\lambda}_2\hfill & \hfill -{\lambda}_2-{\mu}_2\hfill & \hfill {\mu}_2\hfill \\ {}\hfill 1\hfill & \hfill 1\hfill & \hfill 1\hfill & \hfill 1\hfill \end{array}\right) $$

The availability of the original subsystem is *D*_*m*_ = *C*_0_ + *C*_1_ + *C*_2_, which means that the subsystem is available with a single operational unit. Mean availabilities for the original subsystem and for each alternative for improvement are: *D*_0*m*_ = *C*_0_ + *C*_1_ + *C*_2_, *D*_1*m*_ = *C*_0_ + *C*_1_ + *C*_2_ + *C*_3_ and *D*_2*m*_ = *C*_0_ + *C*_1_ + *C*_2_ + *C*_3_ + *C*_4_; where *C*_*i*_ are the coefficients obtained by solving Equation (24), for each alternative.

The failure and repair rates for this subsystem are the same that in the dialysis subsystem for patients with hepatitis C.

### Dialysis subsystem for chronic patients

The dialysis system for chronic patients comprises fourteen positions with a total of eighteen dialysis machines, sharing the workload equally between them. If necessary the machines from the dialysis subsystem for acute patients may be used as provisional replacements.

The subsystem is considered to have failed when four machines break down.

*λ*_*1*_ and *μ*_*1*_ are defined as the failure and repair rates of each monitor when the presence of a maintenance technician is required. *λ*_*2*_ and *μ*_*2*_ are the failure and repair rates of each monitor when the official technical service is contacted.

Two alternatives for improvement are considered, consisting in adding one or two reserve machines to the available set, in the same operating conditions as the others. *D*_*om*_, *D*_*1m*_ and *D*_*2m*_ are defined as the availabilities for eighteen, nineteen and twenty dialysis machines, respectively, considering their replacement to be immediate.

The Markov graph for this subsystem when the presence of a maintenance technician is required is shown in Fig. 4. When the official technical service is contacted, the resulting Markov graph will be the same but characterised by *λ*_*2*_ and *μ*_*2*_.

**Fig. 5:**
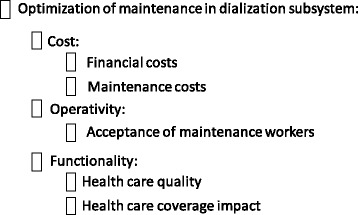
MACBETH'S value tree. Attributes and criteria

The 19 × 19 transition matrix corresponding to the Markov graph when the presence of a maintenance technician is required is shown in Equation (30). The transition matrix corresponding to the case where the official technical service is contacted will be the same as before but characterized by *λ*_*2*_ and *μ*_*2*_.

30 $$ {D}_1=\left(\begin{array}{ccccc}\hfill -14{\lambda}_1\hfill & \hfill {\mu}_1\hfill & \hfill .\hfill & \hfill 0\hfill & \hfill 0\hfill \\ {}\hfill 14{\lambda}_1\hfill & \hfill -13{\lambda}_1-{\mu}_1\hfill & \hfill .\hfill & \hfill 0\hfill & \hfill 0\hfill \\ {}\hfill .\hfill & \hfill .\hfill & \hfill .\hfill & \hfill .\hfill & \hfill .\hfill \\ {}\hfill 0\hfill & \hfill 0\hfill & \hfill .\hfill & \hfill -{\lambda}_1-{\mu}_1\hfill & \hfill {\mu}_1\hfill \\ {}\hfill 1\hfill & \hfill 1\hfill & \hfill .\hfill & \hfill 1\hfill & \hfill 1\hfill \end{array}\right) $$

The availabilities for the original subsystem and for each of the improvement alternatives are: *D*_0*m*_ = *C*_0_ + *C*_1_ + *C*_2_ + *C*_3_, *D*_1*m*_ = *C*_0_ + *C*_1_ + *C*_2_ + *C*_4_ and *D*_2*m*_ = *C*_0_ + *C*_1_ + *C*_2_ + *C*_3_ + *C*_5_; where *C*_*i*_ are the coefficients obtained by solving Equation (24) for each alternative.

### Dialysis subsystem for acute patients

The dialysis subsystem for acute patients has three positions for patients needing temporary dialysis. It comprises five dialysis machines, which share the workload equally between them, so that all work the same number of hours per year. It is not possible to share use with any other subsystem. If necessary, the machines from the dialysis subsystem for acute patients may be used as provisional replacements. The system is considered to have failed when two dialysis machines break down.

*λ*_*1*_ and *μ*_*1*_ are defined as the failure and repair rates of each monitor when the presence of a maintenance technician is required. *λ*_*2*_ and *μ*_*2*_ are the failure and repair rates of each monitor when the official technical service is contacted.

Two alternatives are considered for improving availability, consisting in adding one or two machines to the set available, in the same operating conditions as the others, where *D*_*om*_, *D*_*1m*_ and *D*_*2m*_ are the availabilities for five, six and seven dialysis machines respectively.

The Markov graph and the transition matrix are similar to those obtained with the dialysis subsystem for infectious patients with hepatitis B.

The mean availabilities for the original subsystem, and considering the improvement alternatives are respectively: *D*_0*m*_ = *C*_0_ + *C*_1_ + *C*_2_, *D*_1*m*_ = *C*_0_ + *C*_1_ + *C*_2_ + *C*_3_ and *D*_2*m*_ = *C*_0_ + *C*_1_ + *C*_2_ + *C*_3_ + *C*_4_; where *C*_*i*_ are the coefficients obtained from solving the system of equations in Equation (24) for each alternative.

### Selecting maintenance policies using MACBETH

The working of the subsystems analysed will be described in terms of Markov chains in continuous time, giving systems of equations which provide mean availability values foreseeable over the period of maturity of the life-cycle of each subsystem. Markov chains allow the development of improvement plans for a combination of maintenance policies to be applied to each subsystem, which finally make up the alternatives to be assessed via multicriteria models detailed for each subsystem, and built by MACBETH.

The multicriteria decision making approach described will be applied to a variety of critical subsystems directly related to patient care, and therefore handled by clinical staff. Next, the paper sets out the multicriteria approach developed in four critical subsystems due to the great impact they have on the activity of the hospital; these are the dialysis subsystems of patients infected with hepatitis C and B, in both acute and chronic patients.

### Structuring

Implementation of the MACBETH model proceeds by interviewing a multidisciplinary decision group to obtain scales of attractiveness *v*_*i*_ and weights *w*_*i*_. In this research, the decision-making group is made up of those in charge of the areas of facilities maintenance, maintenance of medical equipment, health and safety, environmental matters, admissions-programming and care staff (comprising those in charge of clinical services and the supervisors of medical areas) of the UGHCR.

The decision-making group was coordinated by the person in charge of technical services at UGHCR, whose responsibilities include the maintenance, safety and environmental departments. The same person also acted as analyst, being knowledgeable about the application of different multicriteria techniques, MACBETH among them. Nominal group techniques were applied to obtain consensus based on structured group discussion [63].

To choose criteria, the decision-making group, and in particular the head of technical services at UGHCR, had information about the decision criteria used in the literature mentioned above; nevertheless, the group chose to use specific criteria adapted to its needs. The criteria used in this research are therefore original and specific to UGHCR, although they may be useful or adaptable to other healthcare organizations.

The decision-making group considered that there are criteria common to all the equipment and others that are specific to the medical device analysed. And so it was necessary to analyse each subsystem individually and in detail. A descriptor was associated with each criterion or subcriterion to make an operational description. A descriptor is an ordered set of plausible performance levels (quantitative or qualitative) to describe the impacts of alternatives with respect to one criterion objectively [42]. A meeting was programmed with the decision-making group for each criterion, and the criteria were classified into economic, operational and functional criteria. A performance scale was produced for each descriptor; to do this, firstly two reference levels were defined: neutral (N), considered by the decision-making group to be neither a satisfactory nor unsatisfactory level, and good (G), considered to be a fully satisfactory level [41]; then additional levels were included, to cover the plausible range of performances and each performance level was described to provide unambiguous interpretation of its meaning at later times.

Economic criteria consider all the annual costs associated with each suggested alternative. The economic criteria considered are:

- Maintenance costs (MCOST). These are the annual maintenance costs of labour and materials associated with a given alternative.
- Financial costs (FCOST). These are annual servicing of debt on the investments made: purchase, installation and booting.

The scale levels of the quantitative descriptors associated with the previous criteria are specific to each subsystem as they depend on the technology that each uses.

The operational criteria are related to the working of the subsystem from the point of view of health professionals or maintenance of the medical equipment. The relevant criterion is:

- Level of acceptance by staff (ACCEP). This measures the level of technical suitability of the working of the subsystem. The aspects considered in the criterion are certainty in giving breakdown diagnoses and the degree of programming of the corrective work. The scale levels of the descriptor used for each criterion are common to the four dialysis subsystems analysed (see Table 2).

**Table 2:** Descriptors and performance levels. The performance levels are ordered in decreasing order of relative attractiveness

Criteria	Descriptors and performance levels	
Financial costs	Annual financial costs required to set up an alternative	
	L1 = Good	0 €
	L2	1.800 €
	L3 = Neutral	3.600 €
	L4	5.400 €
	L5	7.200 €
Maintenance costs	Annual maintenance costs required to set up an alternative	
	L1	10.000 €
	L2 = Good	20.000 €
	L3	30.000 €
	L4 = Neutral	40.000 €
	L5	50.000 €
Degree of acceptance among maintenance personal	Breakdown diagnosis and corrective activity planning capacity	
	L1 = Good	The professional is confident of the diagnoses of the breakdowns analysed. corrective action can be programmed jointly with other subsystems involved.
	L2	The professional is confident of the diagnoses of the breakdowns analysed, and corrective action in the subsystem must be programmed.
	L3 = Neutral	The professional is confident of the diagnoses of the breakdowns analysed, and corrective action in the subsystem must be started immediately.
	L4	The professional is not always confident of the diagnoses of the breakdowns analysed, passing on to his superiors the decision to take corrective or immediate action in the subsystem.
	L5	The professional is not confident of the diagnoses of the breakdowns analysed, and corrective action in the subsystem must be started immediately.
Quality of healthcare	Mean availability of the subsystem and consequences for the working of the subsystem (and so for patient service).	
	L1 = Good	Mean availability of the subsystem is greater than 0.9990. There are no consequences for the working of the subsystem.
	L2	Mean availability of the subsystem is between 0.9990 and 0.9981. A short pause is created in some dialysis posts, with no need to halt the process.
	L3 = Neutral	Mean availability of the subsystem is between 0.9971 and 0.9980. A halt is produced in some dialysis positions, requiring the machines in a normal working state to be stopped, and connected to manual operation and supervised by clinical staff, until they can be returned to automatic operation.
	L4	Mean availability of the subsystem is between 0.9961 and 0.9970. A pause is produced in some dialysis positions, requiring the machines to be disconnected and dialysis to be stopped until normal operation is resumed.
	L5	Mean availability of the subsystem is below 0.9960. A stoppage of the subsystem is produced, implying a 100 % cancellation of the work programmed. A halt is produced in some dialysis positions, requiring the process to be stopped completely.
Impact on care cover	Ability to provide service on a normal working day to other clinical areas or hospitals as required, above the normal work programme.	
	L1	The subsystem allows dialysis sessions to be carried out on patients from other clinical areas, up to a 100 % increase in normal capacity in a normal working day.
	L2 = Good	The subsystem allows dialysis sessions to be carried out on patients from other clinical areas, up to a 100 % increase in normal capacity in a normal working day, and up to 50 % of normal capacity outside normal working hours.
	L3	The subsystem allows dialysis sessions to be carried out on patients from other clinical areas, up to a 50 % increase over normal capacity outside normal working hours.
	L4 = Neutral	The subsystem allows dialysis sessions to be carried out on patients from other clinical areas at certain times, up to an increase of 20 % over normal capacity outside normal working hours.
	L5	The subsystem does not have the capacity to carry out dialysis sessions on patients not included in the normal programme.

The functional criteria are related to the operation of the subsystem; the criteria established by the decision-making group are:

- Quality of care (QUAL). This measures the impact on patient care, as a function of the mean availability of each alternative considered. The scale levels associated with this criterion are established depending on the nature of each subsystem.
- Impact on care coverage (COVER). This shows the possibilities of the subsystem with respect to supply of services, both in the hospital and in other hospitals within its area of influence. The scale levels depend on each subsystem.

The value tree with its hierarchical structure is shown in Fig. 5.

**Fig. 6:**
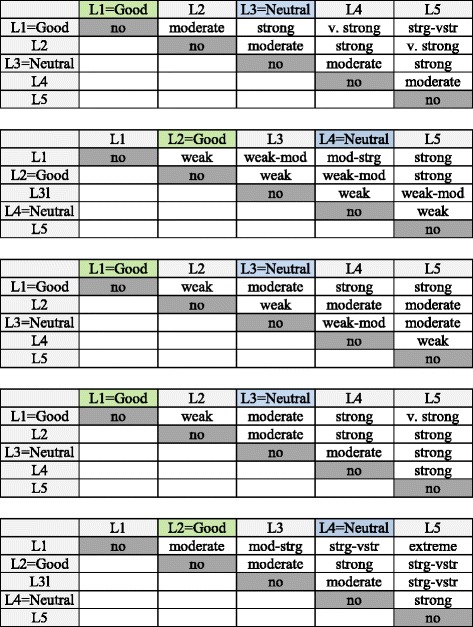
MACBETH Judgement matrix for the criteria of financial costs, maintenance costs, degree of acceptance by staff, quality of healthcare and impact on care cover (from top to bottom)

Table 2 shows the descriptors and scale levels associated with the different criteria in the dialysis subsystems analysed.

### Weighting

A value functions is required to assign value scores to the performance levels of a descriptor relative to the fixed scores of 0 and 100 assigned to the good and neutral reference levels.

To construct a value function for the financial cost criterion, the facilitator (the manager of the maintenance department) asked the decision-making group to judge the differences in attractiveness between the performance levels of the descriptor (see Table 1). Half of the decision-making group considered that the difference between L1 (most preferred level) and L5 (least preferred level) was strong and the other half judged that difference to be very strong. Therefore, the judgement entered in the matrix was strong or very strong. Next, the decision-making group was asked to assess the difference between L2 and L5 levels, unanimously giving a value of very strong. The decision-making group was then asked repeatedly until the last column of the judgement matrix at the top of Fig. 6; next: the first row of the matrix, the diagonal above the main diagonal and the remaining judgements, were completed. With each judgement included in the matrix, M-MACBETH software tested the consistency of all the judgments already formulated and pointed out any situations of inconsistency; it also suggests the minimum number of changes in the judgements needed to solve the problem. The same process is applied to each criterion. Figure 6 shows the final matrices of the qualitative judgments of the group. These judgement matrices were processed in M-MACBETH by means of linear programming to build a value function for each criterion.

**Fig. 7:**
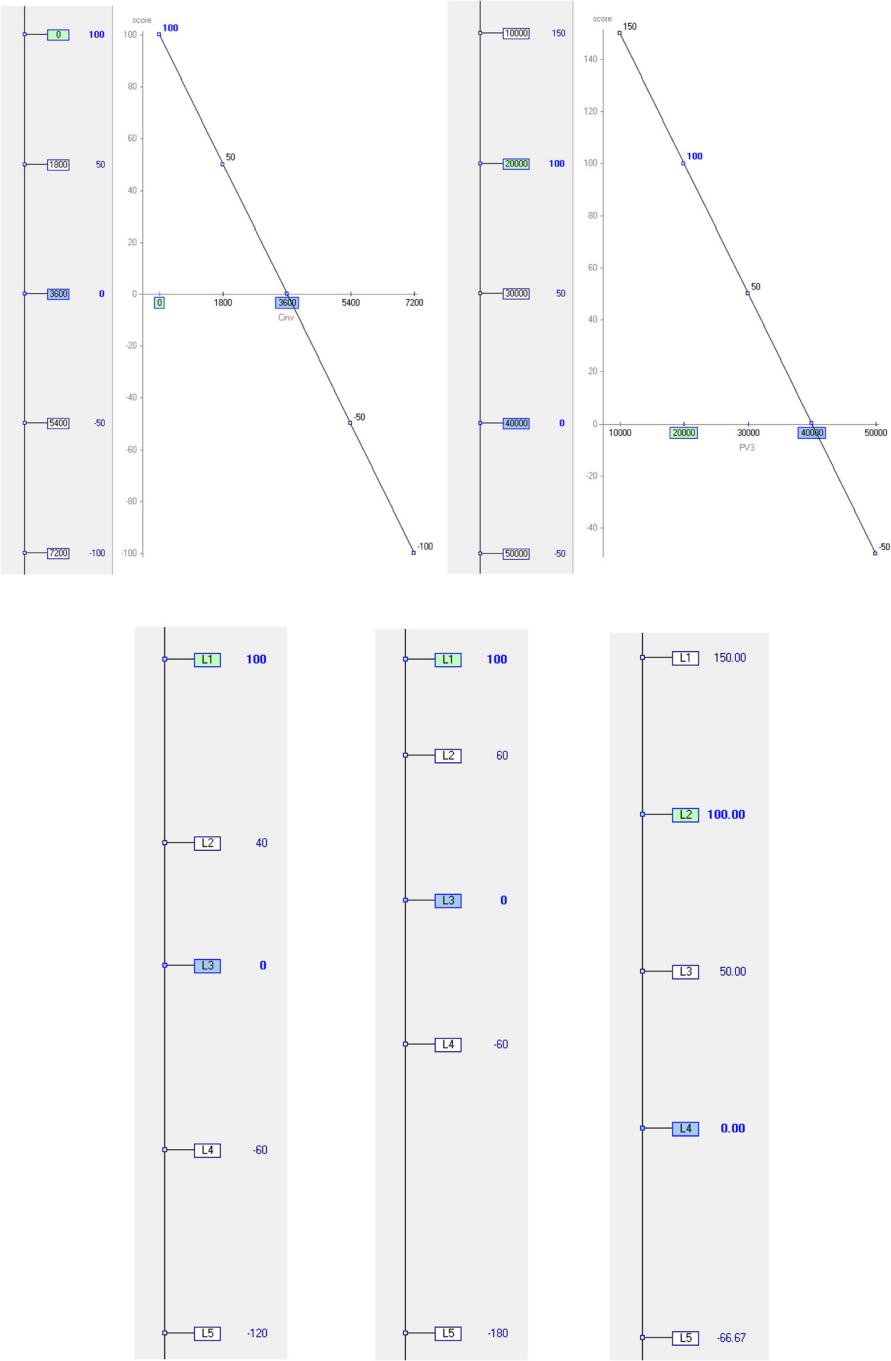
Value functions of criteria: financial costs, maintenance costs, degree of acceptance by staff, quality of healthcare and impact on care cover (from left to right and from top to bottom)

Figure 7 shows the resulting value functions normalized with the neutral level at 0 and the good reference level at 100. In the quantitative criteria, that is, financial and maintenance cost, M-MACBETH provides two graphical displays: a MACBETH scale graph in which each proposed score is plotted at the same point as the respective quantitative performance level and, a piecewise-linear value function graph used to calculate the score of any option whose performance with respect to the criterion is between consecutive performance levels.

Next, the decision-making group assessed the cardinality of the value functions, analysing the proportions of the resulting scale intervals to ensure that their relative size correctly captured the value judgments of the decision-making group; however, no changes in the original value functions were proposed by the group.

**Fig. 8:**
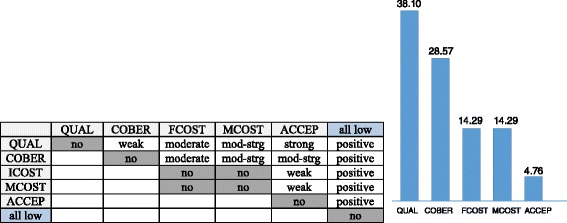
MACBETH judgment matrix for the criteria

The weights were assessed using the MACBETH weighting procedure explained in the MACBETH methodology subsection. The weights obtained are shown in the bar chart of Fig. 8.

### Alternatives

Unlike in most of the literature (see for example [15] and [64]), this paper distinguishes maintenance policy from maintenance strategy. Maintenance strategy is defined as a series of integrated decisions expressed in four structural and six infrastructure decision elements [65].

Different types of maintenance policy can be defined:

- Corrective maintenance, carried out after a failure in order to return the machine to a state in which it can perform its intended function [66].
- Preventive maintenance, carried out at fixed intervals over the working life of the machine, with the aim of reducing the probability of failure or wear of a machine [66].
- Condition Based Maintenance (CBM) or Predictive Maintenance (PDM) is based on the control of physical parameters like vibrations, temperature, particle content of lubricant, etc. in an operational (working) machine, that can be registered, periodically or continuously, by a set of sensors, to detect abnormalities or failures, allowing necessary maintenance activities to be carried out before any catastrophic failure occurs [15].

The Markov chains obtained previously leads to alternative possibilities for combining maintenance policies applied to a subsystem which include: the alternative currently used in the UGHCR, and the two optimization alternatives for the current maintenance policy used by the UGHCR in this subsystem. The following alternatives are considered:

- Corrective and preventive maintenance, with the physical presence of a technician in each shift, for the four subsystems (CM + PM + PPT). This is the alternative currently used by UGHCR. This corresponds to the Markov graphs of Figs. 2, 3 and 4 for the different subsystems. The results are derived from *λ*_*1*_ and *μ*_*1*_.
- Corrective and preventive maintenance plus a reserve machine (CM + PM + 1SP). This action has a similar effect to the former, plus the possibility of substituting a failed monitor for a working one, with zero estimated replacement time. This case applies when the official technical service is contacted, and so the results are derived from *λ*_*2*_ and *μ*_*2*_.
- Corrective and preventive maintenance plus two reserve machines (CM + PM + 2SP) in the same conditions as the previous case, but there now exists the possibility of substituting two failed monitors for other working ones, with zero replacement time. This case applies when the official technical service is contacted, and so the results are derived from *λ*_*2*_ and *μ*_*2*_, but unlike in the previous case there are now two reserve machines instead of one.

### Ranking of alternatives

The evaluation of an alternative is carried out by applying Equation (2) [52].

To assess the alternatives in the criterion quality of care, it is necessary to know the mean availability of each dialysis subsystem for each alternative. Table 3 shows the mean availability obtained for each alternative.

**Table 3:** Mean availability of each subsystem and alternative

Subsystem	Alternatives		
	MC + MP+ PPT	MC + MP + 1SP	MC + MPR + 2SP
Dialysis of patients infected with hepatitis C	0.9977	0.9963	0.9977
Dialysis of patients infected with hepatitis B	1.0000	0.9932	1.0000
Dialysis of chronic patients	0.9999	0.9976	0.9999
Dialysis of acute patients	1.0000	0.9982	1.0000

The result of assessing the impact of the alternatives in the different decision criteria are shown in Table 4.

**Table 4:** Performance of alternatives in the dialysis subsystems

Alternatives	Financial costs (€)	Maintenance costs (€)	Degree of acceptance among staff	Quality of healthcare	Impact on care coverage
Subsystem for dialysis of patients infected with hepatitis C					
CM + PM+ PPT	0	21,000	L1	L3	L4
CM + PM + 1SP	1,800	16,000	L2	L4	L3
CM + PM + 2SP	3,600	18,000	L2	L1	L3
Subsystem for dialysis of patients infected with hepatitis B					
CM + PM+ PPT	0	15,000	L1	L1	L4
CM + PM + 1SP	1,800	12,000	L2	L3	L2
CM + PM + 2SP	3,600	12,000	L2	L1	L2
Subsystem for dialysis of chronic patients					
CM + PM+ PPT	0	54,000	L1	L1	L5
CM + PM + 1SP	1,800	38,000	L3	L3	L4
CM + PM + 2SP	3,600	40,000	L2	L1	L4
Subsystem for dialysis of acute patients					
CM + PM+ PPT	0	15,000	L1	L1	L4
CM + PM + 1SP	1,800	12,000	L2	L3	L2
CM + PM + 2SP	3,600	14,000	L2	L1	L2

## Results

**Fig. 9:**
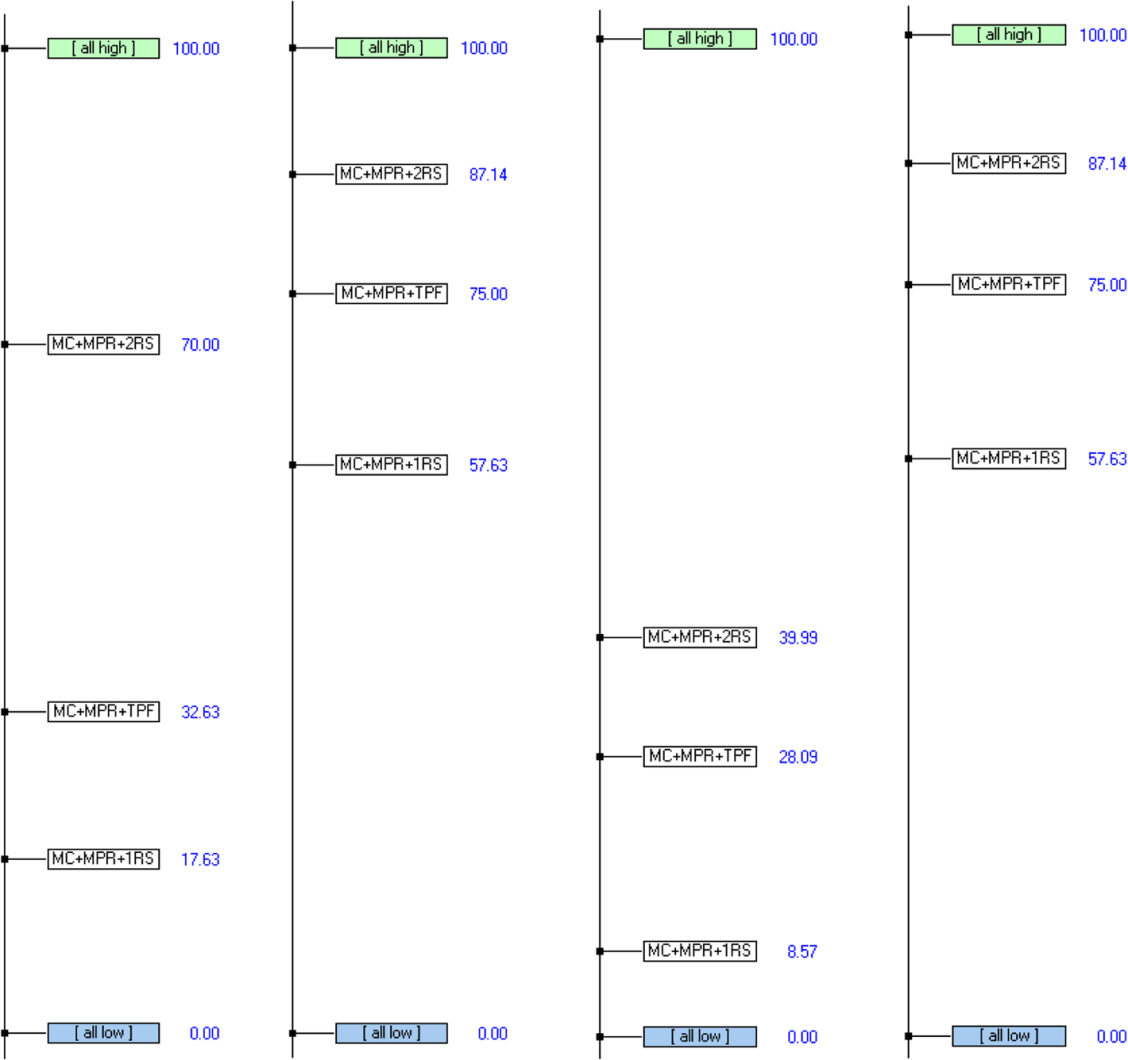
Overall ranking of alternatives for dialysis subsystems: for patients with hepatitis C, patients with hepatitis B, chronic patients, and acute patients (from left to right)

Figure 9 shows the classification of the alternatives obtained for the different dialysis subsystems. It can be seen that in all cases the maintenance strategy obtained consists of applying corrective and preventive maintenance plus two reserve machines (CM + PM + 2SP).

**Fig. 10:**
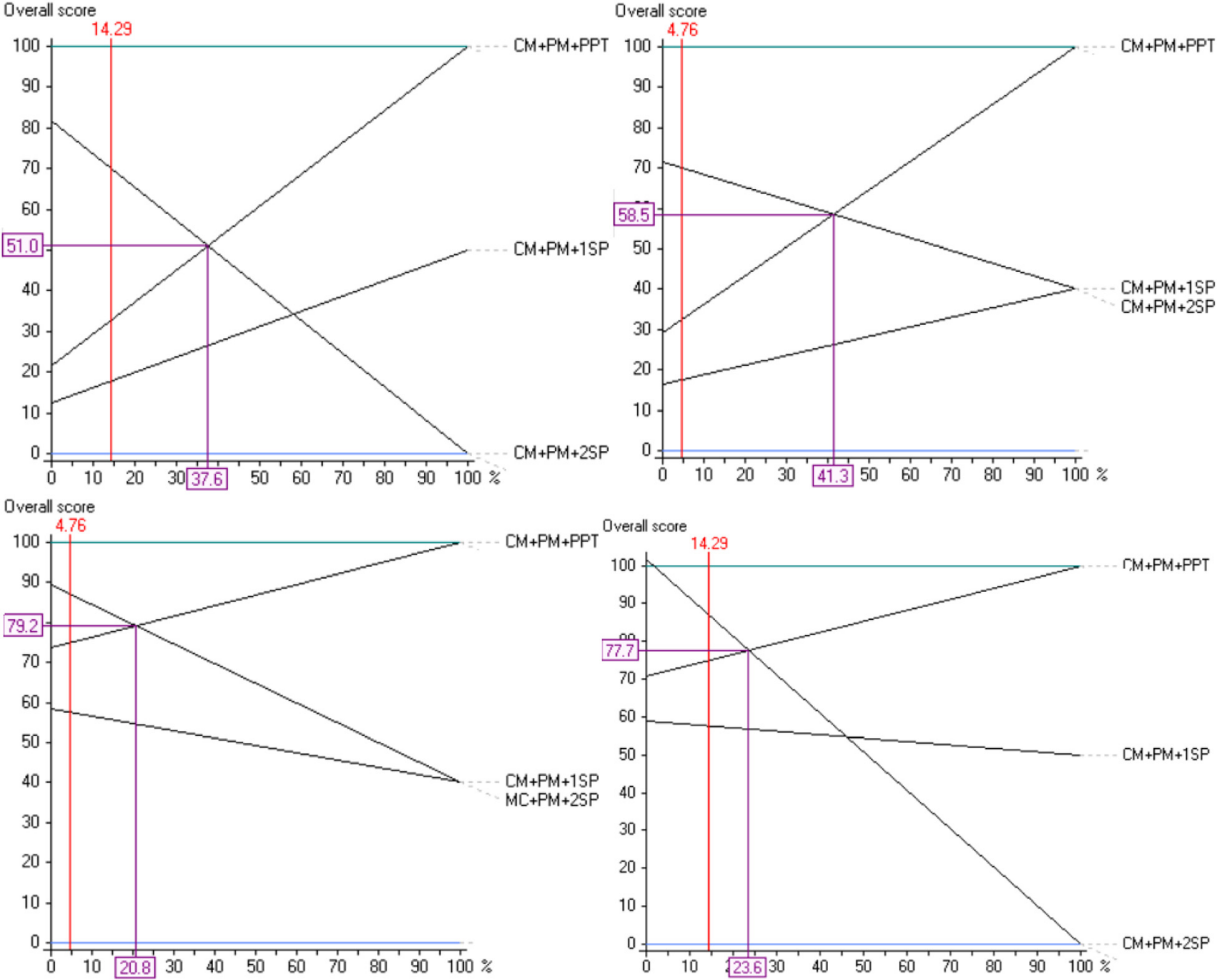
Results of the sensitivity analysis (from left to right and from top to bottom): Dialysis subsystem for patients with hepatitis C (financial costs), dialysis subsystem for patients with hepatitis C (degree of acceptance among staff), dialysis subsystem for patients with hepatitis B (degree of acceptance among staff), dialysis subsystem for acute patients (financial costs)

Next, a sensitivity analysis was performed to assess the implications of a logical modification of the weightings of given decision criteria. The alternatives proposed for the multicriteria approach do not necessarily involve an improvement in functionality and of the quality of care. The weightings of the criteria with minimal impact on quality of care have therefore been modified, that is the cost criteria and the level of acceptance among the staff. Figure 10 shows, as an example, several results of the sensitivity analysis. The vertical red line represents the current weighting of the criterion analysed. The violet line shows the weight associated with the intersection of two alternatives and, therefore, the weighting necessary to swap their rank in overall attractiveness. In all cases a significant increase would be necessary in the weightings of the criteria, which would be illogical according to the judgements given, to cause a change in the classification of the first-placed alternative.

Table 5 shows the costs, their consequences for care and availability of the alternatives currently applied in the subsystems analysed in the UGHCR and those obtained as ideal solutions by the multicriteria approach described. From the point of view of maintenance, not assigning fixed resources to technicians for dialysis maintenance, that is, a change from the alternative CM + PM + PPT to the alternative suggested by the multicriteria approach, CM + MP + 2SP, involves:

**Table 5:** Maintenance policy currently applied in the UGHCR and the best valued by the multicriteria approach

Subsystem	Alternative currently applied				Alternative provided by this research			
	Description (value)	Costs (€)	Consequences for care	Availability	Description (value)	Costs (€)	Consequences for care	Availability
Dialysis of patients with hepatitis C	CM + PM+ PPT (32.63)	Mainte-nance cost: 21.000 Financial cost: 0	The patient suffers no decrease in quality of service once the dialysis has begun. Increases the possibility of carrying out unprogrammed dialysis motivated by the admission of a patient for other reasons but who requires, for an unexpected reason, sporadic dialysis (increase in supply in intrahospital emergency situations)	0.9977	CM + PM + 2SP (70.00)	Mainte-nance cost: 18.000 Financial cost: 3.600	The patient suffers no decrease in quality of service once the dialysis has begun. Increases the possibility of carrying out unprogrammed dialysis motivated by the admission of a patient for other reasons but who requires, for an unexpected reason, sporadic dialysis, from the same hospital or from another hospital within the catchment area (increase in supply in intrahospital and extrahospital emergency situations). Increase the supply emergency hospital situations and programmed actions within the catchment area	0.9977
Dialysis of patients with hepatitis B	CM + PM+ PPT (75.00)	Mainte-nance cost: 15.000 Financial cost: 0	The patient suffers no decrease in quality of service once the dialysis has begun. Increases the possibility of carrying out unprogrammed dialysis motivated by the admission of a patient for other reasons but who requires, for an unexpected reason, sporadic dialysis (increase in supply in intrahospital emergency situations)	1	CM + PM + 2SP (87.14)	Mainte-nance cost: 14.000 Financial cost: 3.600	The patient suffers no decrease in quality of service once the dialysis has begun. Increases the possibility of carrying out unprogrammed dialysis motivated by the admission of a patient for other reasons but who requires, for an unexpected reason, sporadic dialysis, from the same hospital or from another hospital within the catchment area (increase in supply in intrahospital and extrahospital emergency situations).	1
Dialysis of chronic patients	CM + PM+ PPT (28.09)	Mainte-nance cost: 54.000 Financial cost: 0	The patient suffers no decrease in quality of service once the dialysis has begun	0.9999	CM + PM + 2SP (39.99)	Mainte-nance cost: 40.000 Financial cost: 3.600	The patient suffers no decrease in quality of service once the dialysis has begun. Increases the possibility of carrying out unprogrammed dialysis motivated by the admission of a patient for other reasons but who requires, for an unexpected reason, sporadic dialysis, from the same hospital or from another hospital within the catchment area (increase in supply in intrahospital and extrahospital emergency situations)	0.9999
Dialysis of acute patients	CM + PM+ PPT (75.00)	Mainte-nance cost: 15.000 Financial cost: 0	The patient suffers no decrease in quality of service once the dialysis has begun. Increases the possibility of carrying out unprogrammed dialysis motivated by the admission of a patient for other reasons but who requires, for an unexpected reason, sporadic dialysis (increase in supply in intrahospital emergency situations)	1	CM + PM + 2SP (87.14)	Mainte-nance cost: 14.000 Financial cost: 3.600	The patient suffers no decrease in quality of service once the dialysis has begun. Increases the possibility of carrying out unprogrammed dialysis motivated by the admission of a patient for other reasons but who requires, for an unexpected reason, sporadic dialysis, from the same hospital or from another hospital within the catchment area (increase in supply in intrahospital and extrahospital emergency situations).	1

- The time assigned to human resources in maintenance dedicated exclusively to the dialysis subsystems is no longer required, and can be reassigned to other activities. Therefore, efficiency increases and global costs decrease.
- Overspecialization of human resources in this type of medical equipment is eliminated, allowing a group of versatile technicians to be created to cover all subsystems, as long as the same strategy is used for each subsystem.

In the dialysis subsystems, when there is an improvement in quality of care, and the supply of patient attention is broadened, a plan has been devised for introducing improvements in maintenance policies, considering the costs associated with them. To introduce the best alternatives the following priorities have been established:

- Priority 1: Actions that provide direct improvement in subsystem functionality.
- Priority 2: Actions that improve the maintainability of the subsystems.
- Priority 3. Actions obtained by modifying the specific weighting of some criterion.

The four subsystems analysed have priority 1 for the introduction of the alternatives obtained in from the study. In the four subsystems analysed the time required to introduce a CM + PM + 2SP policy has been estimated at 1 month; it is felt that normal activities carried out by maintenance personal on the subsystems will not change.

## Discussion and conclusions

The multicriteria decision-making approach presented in this research includes the use of Markov chains, which have allowed the mean availability over the useful life of each of the assets studied, grouped into subsystems, to be obtained, and maintenance policies and possible improvements to be defined, that ultimately represent the alternatives. The previous data have been used in a specific multicriteria decision model for each subsystem, in which a multi-disciplinary decision-making group at the hospital took part, so as to guarantee applicability. In the models constructed via the MACBETH approach, economic criteria have been used, related to the quality of care which is desired for patients (availability), and to the acceptability and acceptance that each alternative would have considering the maintenance and healthcare resources which exist in the organization. Since the users of medical devices (doctors, nurses, clinical technicians, etc.), and not only maintenance workers, have an important influence on the availability of systems, their inclusion in the decision-making group clearly sets this research apart from other studies.

There is currently no similar multicriteria approach in the literature related to the optimization of maintenance, through which the maintenance policies or action strategies considered standard in the UGHCR have been defined, and using these as a starting point, others with higher specifications have been considered. Given that the hospital has innumerable, highly varied, subsystems, from the functional and technological point of view, the criteria, impact levels and alternatives considered are very different in each. This paper applies the multicriteria approach to the dialysis subsystems for patients infected with hepatitis C and B, both chronic and acute patients. These subsystems have the peculiarity that they interact directly with patients, and for this reason they are critical. The specific characteristics of other subsystems mean that both the alternatives and the decision criteria of the multicriteria model are different from the judgements of the decision-making group.

Unlike most contributions in the literature, this paper analyses alternatives that are a combination of maintenance policies and other additional improvements, instead of independent maintenance policies. The multicriteria approach is therefore better suited to actual business practice, since industry habitually applies this combination of maintenance policies.

It should be pointed out that in the alternatives, and depending on the subsystem analysed, improvements are introduced that are not included in normal maintenance policies, involving broader actions and with specifically measurable results, such as: availability of new active or reserve equipment, specific spares, increase in physical presence, outsourcing of the function or service, etc. Thus the alternative suggested for each subsystem offer real, clearly distinguished, solutions. In this way, not only have different maintenance policies been suggested, but also alternatives that, in each case and according to viability, provide a more complete decision tool for the maintenance manager.

As future research, the idea is to control the introduction of the suggested alternatives. To this end an introduction plan has been drawn up which identifies the priority for the adaptation of new maintenance policies to the existing ones in the hospital, by subsystem. This plan is expected to be carried out in the long term. The hope is to control how the change from the currently-used alternative to the alternative proposed by the model actually affects the costs, availability, safety, the environment and especially, quality of health care.

Furthermore, the models proposed for each subsystem should be regularly updated, either because of the increase in the amount of medical equipment which increases the demand for spaces and reserve equipment, modifying or increasing the proposed alternatives; or because of the need to review the decision criteria included in the model, in view of new characteristics or healthcare needs.

### Availability of data and materials

The data from this research are maintained by the Technical Services of the General Hospital of Ciudad Real. These data were obtained and calculated by Andrés Gómez Blanco, Vice-director of Technical Services of the University General Hospital of Ciudad Real. The specific data used in this study is available upon request from the authors.
